# Role of respiratory intermediate care units during the SARS-CoV-2 pandemic

**DOI:** 10.1186/s12890-021-01593-5

**Published:** 2021-07-13

**Authors:** Mónica Matute-Villacís, Jorge Moisés, Cristina Embid, Judith Armas, Isabel Fernández, Montserrat Medina, Miquel Ferrer, Oriol Sibila, Joan Ramón Badia

**Affiliations:** 1 Servei de Pneumologia i Al·lèrgia Respiratòria, Institut Clínic Respiratori, Hospital Clínic de Barcelona, Universitat de Barcelona, IDIBAPS, Barcelona, Spain; 2 CIBER de Enfermedades Respiratorias (CIBERES), Madrid, Spain

**Keywords:** COVID-19, SARS-CoV-2, RICU, Intermediate care, Intensive care, Respiratory failure, Tracheostomy

## Abstract

**Rationale:**

The SARS-CoV2 pandemic increased exponentially the need for both Intensive (ICU) and Intermediate Care Units (RICU). The latter are of particular importance because they can play a dual role in critical and post-critical care of COVID-19 patients. Here, we describe the setup of 2 new RICUs in our institution to face the SARS-CoV-2 pandemic and discuss the clinical characteristics and outcomes of the patients attended.

**Methods:**

Retrospective analysis of the characteristics and outcomes of COVID-19 patients admitted to 2 new RICUs built specifically in our institution to face the first wave of the SARS-CoV-2 pandemic, from April 1 until May 30, 2020.

**Results:**

During this period, 106 COVID-19 patients were admitted to these 2 RICUs, 65 of them (61%) transferred from an ICU (step-down) and 41 (39%) from the ward or emergency room (step-up). Most of them (72%) were male and mean age was 66 ± 12 years. 31% of them required support with oxygen therapy via high-flow nasal cannula (HFNC) and 14% non-invasive ventilation (NIV). 42 of the 65 patients stepping down (65%) had a previous tracheostomy performed and most of them (74%) were successfully decannulated during their stay in the RICU. Length of stay was 7 [4–11] days. 90-day mortality was 19% being significantly higher in stepping up patients than in those transferred from the ICU (25 vs. 10% respectively; p < 0.001).

**Conclusions:**

RICUs are a valuable hospital resource to respond to the challenges of the SARS-CoV-2 pandemic both to treat deteriorating and recovering COVID-19 patients.

## Introduction

The outbreak of a novel coronavirus SARS-CoV2 causing COVID-19 (coronavirus disease 2019) has led to an unprecedented international health crisis. On March 11^th^, the World Health Organization (WHO) declared a global pandemic due to the rapid increase in the number of cases outside China. Since then, healthcare response to the COVID-19 pandemic has been a major concern for public health services and nations around the world [1].

The high transmissibility of the SARS-CoV-2 [2] and the fact that 5–15% of all infected patients will develop severe COVID-19 disease rapidly filled up the available Intensive Care Unit (ICU) beds [3] and led to contingency plans to increase their number by using other ICU beds, such as those normally dedicated to post-operative support (with a parallel reduction in surgical activity) and even to the conditioning of the operating rooms themselves to provide critical care to severe COVID-19. In this scenario, Respiratory Intermediate Care Units (RICU) played an important double role. First, by facilitating the step-down of ICU patients (hence reducing their length of stay in ICU which, in turn, facilitated the care of new critically ill patients), many of them with tracheostomy and ICU-associated myopathy that require expert care including rehabilitation [4–6]. Second, by providing high-flow oxygen therapy via nasal cannula (HFNC) or non-invasive ventilation (NIV) in less severe patients (who may eventually require ICU care too (step-up)) or in those who may not be candidates for mechanical ventilation due to concomitant conditions [7].

Here, we: (1) describe the setup of 2 new RICUs in our institution to face the SARS-CoV-2 pandemic; and, (2) discuss the clinical characteristics and outcomes of the patients attended there.

## Methods

### Organizational RICU aspects

To respond to the increased health-care demands caused by the first wave of the SARS-CoV-2 pandemic, our institution built/transform several new ICU and RICUs. We describe here the logistics and organization of two of them, who were designed, lead and managed by members of the Pulmonary Division of Hospital Clinic (Barcelona, Spain).

### Clinical performance

We analyzed retrospectively the characteristics and outcomes of patients with moderate/severe COVID-19 admitted to these two RICUs from April 1 until May 30, 2020 because: (1) severe respiratory failure with high oxygen requirements (FiO_2_ > 40%); (2) need of ventilatory support with NIV or HFNC (PaO_2_ < 60 mmHg, respiratory rate (RR) > 30 bpm, chest incoordination, respiratory acidosis and/or hypercapnia); (3) septic shock; (4) transferred from ICU; and/or (5) not candidates for admission to ICU (do not resuscitate order). In these patients, we analyzed their anthropometric data, comorbidities, previous treatments, treatment received for COVID-19, length of stay (LOS) and mortality.

### Ethics

The study was approved by our Institutional Review Board (Comité Ètic d´Investigació Clínica – Hospital Clinic de Barcelona. HCB/2021/0425).

### Statistical analysis

Results are expressed as mean ± standard deviation (SD) for quantitative variables that followed a normal distribution, and as median and IQR otherwise. Qualitative variables are expressed as total number and percentage. Fisher exact test was used to compare qualitative variables. Student T-Test or Mann–Whitney U test, as appropriate, were used to compare quantitative variables. Kaplan Meier curves for 90 days mortality was compared by the log-rank test. A two-sided p value lower than 0.05 was considered statistically significant. Analyses were done using SPSS (version 22.0; SPSS Inc, Chicago, Illinois, USA).

## Results

### Organizational aspects

**Fig. 1:**
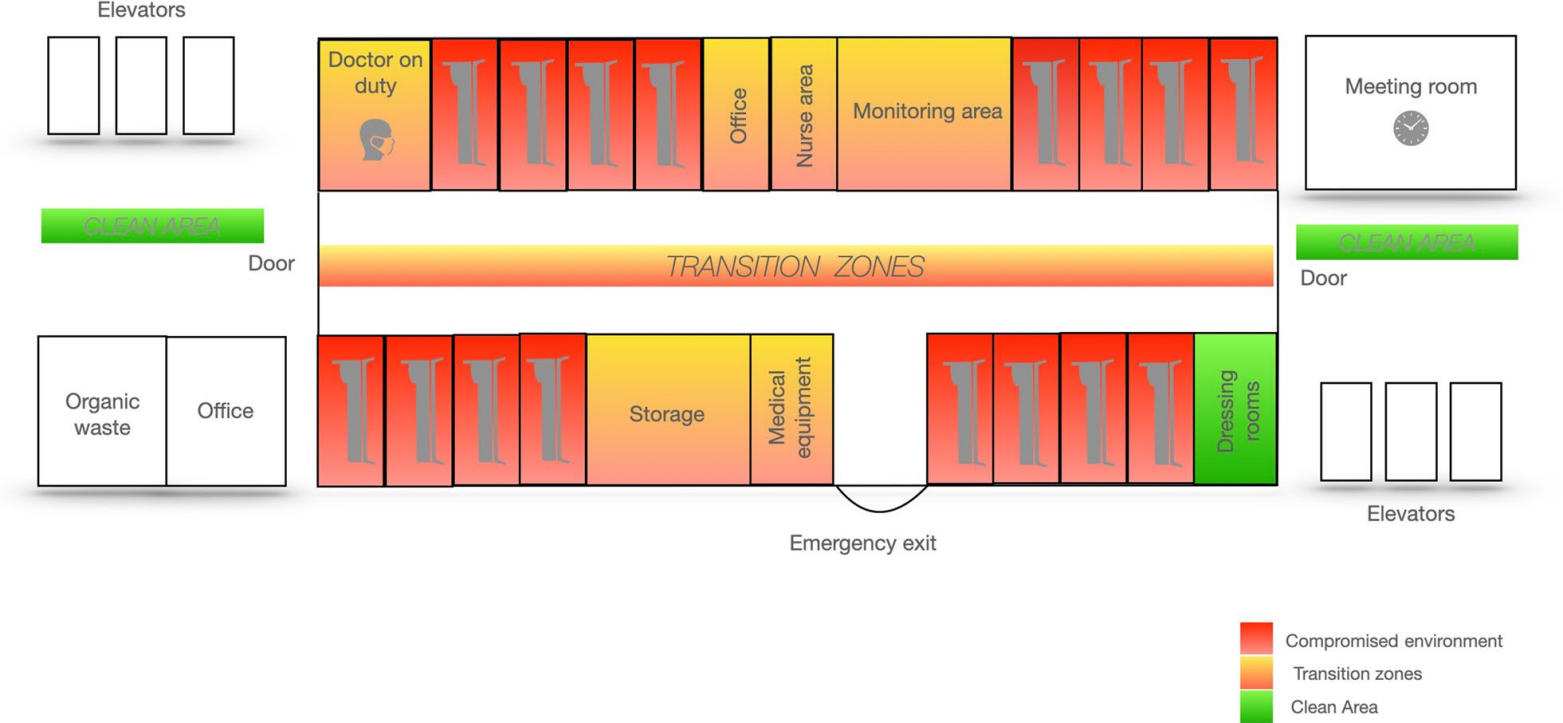
Functional organizational of Unit A. For further explanations, see text

Under normal operational conditions, our institution (Hospital Clínic Barcelona) has 850 conventional ward beds and 44 ICU beds of different medical and surgical specialties. During the first wave of SARS-CoV-2 pandemic, the hospital had to re-engineer additional spaces to care for COVID-19 patients. Our Pulmonary Department was asked to transform 2 conventional wards into 2 RICUs (named here A and B) exclusively devoted to the treatment of critically ill COVID-19 patients: unit A had 16 beds and unit B 10 beds. The functional organization of both RICUs sought to minimize the risk of virus spreading among staff (Fig. 1). All rooms were for individual use, had a private bathroom and a glass door for external visual control. All were equipped with oxygen and air supply, a video system with centralized continuous (non-invasive and invasive if needed) monitoring, but not with negative pressure. Electrocardiogram recording system (KARDIA), a portable ultrasound (General Electric and Phillips). NIV, HFNC and Cardiopulmonary Resuscitation (CPR) equipment’s were available in both units.

**Fig. 2:**
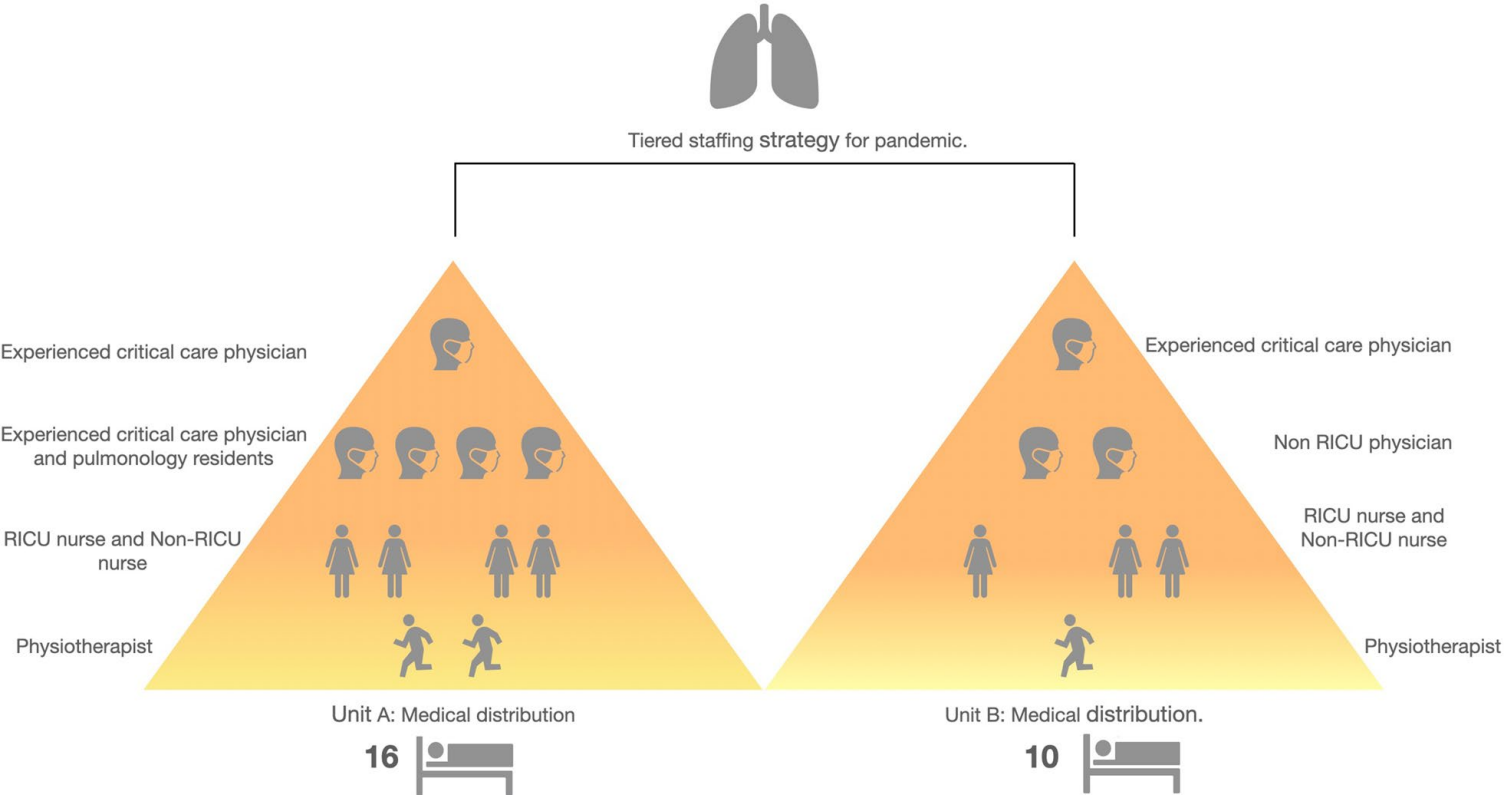
Organizational pyramid of staff in the two RICUs. Modified from the Ontario Health Plan for an Influenza Pandemic Workgroup. For further explanations, see text

The sanitary personnel of needed in each unit was calculated following local [8] and international recommendations[9]. Briefly, the tiered staffing distribution aimed at: (1) achieving a patient/physician ratio of 4/1, and a patient/nurse ratio of 4/1; and, (2) guaranteeing that each unit has, at least, one highly trained critical care physician and one critical care nurse who directly supervise the rest of staff (who may or may be not trained in RICU care previously). As shown in Fig. 2, we assigned to Unit A (16 beds) 2 pulmonologists with critical care expertise, 2 pulmonology residents, 2 nurses with critical care expertise, 2 nurses with previous experience in other areas and 2 respiratory physio-therapists, while in Unit B (10 beds) we assigned 1 pulmonologist with critical care expertise, 4 physicians from other specialties (e.g. nephrology, traumatology, neurology), 1 nurse with critical care expertise, 2 nurses experienced in other clinical areas and 1 respiratory physio-therapist. A pulmonologist was on site in each Unit overnight.

### Clinical performance

#### Patient characteristics

During the study period, a total of 2,238 patients were admitted to our Hospital with COVID-19 infection confirmed by Real-Time Polymerase Chain Reaction (RT-PCR) for SARS-CoV-2 [10], among whom, 475 patients (21.2%) required ICU admission.

Table 1 presents the main clinical characteristics of the 106 patients admitted to one of the two RICU investigated here. Mean age was 66 ± 12 years, most of them (72%) were males with a mean body mass index (BMI) of 29 kg/m^2^. The most prevalent comorbidity was systemic arterial hypertension (54%). The majority of patients (61%) admitted to the 2 RICUs analyzed here were discharged from a hospital ICU (step-down); the rest came from either a hospital ward (26%) or directly from the emergency room (13%). Twenty-eight patients (26%) were admitted with a Do Not Resuscitation (DNR) standing order.

**Table 1:** Clinical characteristics of the 106 patients with COVID-19 admitted to the RICU

*Demographics*	
Age mean (SD)	66 ± 12
Gender male (%)	76 (72%)
Mean body weight, kg mean (SD)	83 ± 17
Body mass index, kg/m^2^ mean (SD)	29 ± 6
*Comorbidity*	
Systemic arterial hypertension	57 (54%)
Dyslipidemia	30 (28%)
Cardiovascular disease	20 (19%)
Chronic obstructive pulmonary Disease	16(15%)
Diabetes	14 (13%)
Chronic renal failure	14 (13%)
Obstructive sleep apnea	10 (9%)
Cerebrovascular disease	7 (6.6%)
Cancer history	6 (6%)
Organ transplantation	5(5%)
Asthma	3(3%)
*Previous treatment*	
Statins	19(18%)
Angiotensin II receptor blockers	9 (8.4%)
*Source of admission to RICU*	
Intensive care unit (step-down)	65 (61%)
COVID-19 ward (step-up)	27 (26%)
Emergency room (step-up)	14 (13%)

Data is presented as mean (SD) or number of patients (%)

Table 2 details the type of treatment received while in the RICU. Medical treatment followed the recommendations at that time which, unfortunately, were not based on evidence. Thirty-three patients (31%) required HFNC, 15 (14%) NIV and 11 (10%) both (HFNC and NIV). NIV was indicated in patients who deteriorated despite HFNC (as indicated by the presence of severe arterial hypoxemia (PaO_2_ < 60 mmHg despite elevated FiO_2_ concentrations (> 80%—60 lpm)), Respiratory Rate (RR) > 30/min, chest incoordination, respiratory acidosis and/or hypercapnia while on HFNC), or who were not candidates for ICU admission due to DNR. The median duration of HFNC and NIV was 5 days [IQR 3–7] and 4 [IQR 1–6.5], respectively. Forty two of the 106 patients (40%) had a tracheostomy when admitted and most of them were decannulated (74%) during their stay in the RICU with a median time from admission to decannulation of 8 [5–12] days.

**Table 2:** Treatments and outcomes

*COVID-19 treatment*	
Hydroxychloroquine	73 (69%)
Systemic corticosteroids	66 (62%)
Azithromycin	60 (57%)
Tocilizumab	51 (48%)
Anakinra	22 (21%)
Siltuximab	5 (5%)
Remdesivir	5 (5%)
*Respiratory support in RICU*	
High flow oxygen nasal cannula	33 (31%)
Non-invasive ventilation	15 (14%)
Days since tracheostomy to decannulation (median [IQR]; n = 31)	25.5 [19–34]
Days since admission to decannulation (median [IQR]; n = 31)	8 [5–12]
NIV duration, days (median [IQR]; n = 14)	4 [1–6.5]
HFNC duration, days (median [IQR]; n = 32)	5 [3–7]
Renal replacement therapy	9 (9%)
Vasopressors	3 (3%)
*Destination*	
Discharge to COVID-19 ward	75 (71%)
Transfer to ICU	17 (16%)
Death	14 (13%)
*Outcomes*	
30-day mortality	15 (14%)
Global mortality at 90 days	20 (19%)
Days since admission to RICU (median [IQR])	13 [4–25.3]
LOS in RICU (median [IQR])	7 [4–11]
LOS (median [IQR])	34 [17–52]
Days since admission to death	27 [12–34]

Data is presented as number of patients (%), median [interquartile range]

*LOS* length of stay (days)

Table 3 compares the clinical characteristics of patients by source of admission (ICU or non-ICU (ward and ER). The latter were older, had a lower BMI and more comorbidities and required more often HFNC, although NIV was used more frequently in patients admitted from an ICU.

**Table 3:** Characteristics of patients by source of admission

	ICU (n = 65)	Non-ICU (n = 41)	p
Age, years, mean	64 ± 11.5	69 ± 13.3	0.048
Body mass index, kg/m^2^, mean (SD)	31 ± 6	27 ± 5	0.002
Comorbidities ≥ 2, n (%)	25 (38.5)	25 (61)	0.029
High flow nasal cannula, n (%)	16 (25)	17 (41)	0.086
Non-invasive ventilation, n (%)	5 (8)	10 (24)	0.022
90-day mortality (%)	16 (25)	4 (10)	< 0.001

Data is presented as mean (SD) or number of patients (%)

#### Outcomes

The length of stay (LOS) was 34 days and the LOS in the RICU was 7 days. Table 2. The majority of admitted patients (71%) were discharged from RICU to a COVID-19 Ward, 16% of them were transferred to an ICU and, unfortunately, 14 patients (13%) died during their RICU stay. 3 patients were transferred to the ICU due to the requirement of orotracheal intubation [2] or reconnection to mechanical ventilation through a tracheostomy tube [1]. The mean time since admission to RICU until death was 27 days [12–34 days]. Two deceased patients came from ICU (step-down) and 12 came from the emergency room (ER) or COVID-19 wards (step-up). Most of the 14 deceased patients (50%) had an indication for DNR. We had no readmissions in our RICUs.

**Fig. 3:**
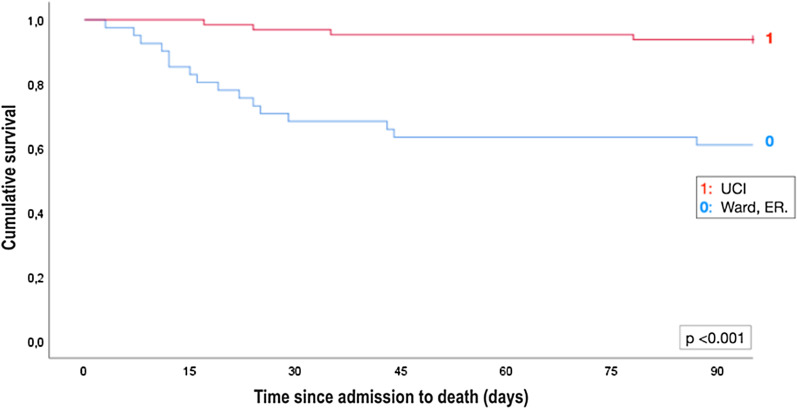
Kaplan Meier 90-day mortality curves in patients admitted to the RICU from an ICU or non-ICU (ward, ER) settings. For further explanations, see text

Patients were followed up for at least 90 days. Mortality at 30 days was 14% (n = 15). Mortality at 90-days was 19% (n = 20) and, as illustrated in Fig. 3, it was higher in patients transferred from ward or ER compared to patients transferred from the ICU (25% vs. 10%, p < 0.001).

## Discussion

This study details the setting and operation of two RICUs created *ex-novo* to care for COVID-19 patients. Operational results show that RICUs are a viable alternative to increase ICU bed availability maintaining high-quality care. This setting can contribute to a faster recovery process providing specifically dedicated physiotherapy and improving patient care by having a higher doctor-patient/nurse-patient ratio than available in a conventional ward while being a less expensive asset in comparison with the ICU [11]. The postcritical COVID-19 patient has a variety of active medical problems becoming a highly demanding patient in terms of specific care. Therefore, RICU provides multidisciplinary care that shortens ICU stay and could potentially shorten overall LOS.

RICUs are a valuable asset for either large or smaller hospitals, providing flexibility and a suitable environment of care for many types of patients and clinical situations. However, RICU is still not implemented in many hospitals, COVID-19 pandemic has highlighted the importance of these units in avoiding hospital collapse. Patients with COVID-19 presents with acute severe respiratory failure requiring ventilatory support and continuous monitoring. Given the immediate saturation of ICU beds that occurred during the first pandemic wave, it was critical and urgent to create units that could cope with large numbers of patients, either requiring high care needs or coming from overloaded ICUs.

During the study period, a 2.5-fold increase in the number of RICU beds was achieved in Spain. In a survey conducted by Caballero et al., 41 centres confirmed that at least one RICU was available, with an overall significant increase in the number of RICU beds from 112 to 525. Regarding staff, 95% of these units had at least 1 specialist in pulmonology either involved o directly in charge [11–13].

In our institution, in a short period of time, we achieved a 6.5-fold increase in the number of RICU beds. This milestone was achieved with the involvement of pulmonologists and other professionals with expertise in respiratory medicine. This background provided solid clinical training in the assessment and treatment of respiratory failure, airway management and the management of respiratory support. However, the key to success is in our view, was teamwork and a multidisciplinary approach involving specialized nurses in respiratory care and respiratory therapists in a highly focused environment to guarantee proper functioning and performance. In addition to having the necessary medical equipment and diagnostic tools [14].

Most of the patients admitted to our RICU came from ICU (61%) and 40% of all (42/106) had a previous tracheostomy performed. Thus, the main role of a RICU during a pandemic was to relieve the high ICU load to allow the high demanding bed’s rate and turn over required under that scenario. The second but not the less is to achieve this target without increasing the risk of related complications because of an early discharge from ICU. RICU as we have described fulfills this function as is shown in terms of 30 and 90-day mortality. Moreover, our purpose during RICU stay was to decannulate all the patients as a mandatory requirement previous the ward discharge to avoid the high risk of cannula complications in a non-monitored ward. The role of respiratory therapists was essential to successfully manage the decannulation process in a short period of time since patients were admitted to our RICU (median 8 [5–12] days). In addition to all the above, the RICUs created were essential to support the 32 wards fully dedicated to caring for COVID patients, both for those worsening in the wards.

In our study, the overall 90-day mortality rate was 18.5%, in contrast with previous publications with slightly higher mortality reported in a different clinical setting, were the majority of patients were included in a step-up setting [15, 16].

We observed that patients transferred from non-ICU departments were older, had more comorbidities, had a lower BMI and had a statistically and clinically relevant higher mortality. This data is likely attributable to the fact that many of the admissions had standing DNR orders. Thus, HFNC and or NIV were considered in some of these cases the maximum level of respiratory support.

In absence of such limitations, we were very active in avoiding delay in intubation or admission to the ICU and the start of invasive mechanical ventilation in those patients who met the criteria for admission to the ICU.

The present study is descriptive and uncontrolled because of the difficulty of comparing our results with other units and historical data in this unprecedented pandemic situation.

In conclusion, the results of this study show that RICUs are valuable in this health care crisis and have a relevant role in terms of acute respiratory patient management. The success of this type of units should be taken into account when considering organizational changes that can prepare the healthcare system for the current ongoing pandemic and future challenges.

## Data Availability

The datasets used and/or analysed during the current study available from the corresponding author on reasonable request.
